# Co-activation: its association with weakness and specific neurological pathology

**DOI:** 10.1186/1743-0003-3-26

**Published:** 2006-11-20

**Authors:** Monica E Busse, Charles M Wiles, Robert WM van Deursen

**Affiliations:** 1 Department of Physiotherapy, Cardiff University, Cardiff, UK; 2 Department of Neurology, Cardiff University, Cardiff, UK

## Abstract

**Background:**

Net agonist muscle strength is in part determined by the degree of antagonist co-activation. The level of co-activation might vary in different neurological disorders causing weakness or might vary with agonist strength.

**Aim:**

This study investigated whether antagonist co-activation changed a) with the degree of muscle weakness and b) with the nature of the neurological lesion causing weakness.

**Methods:**

Measures of isometric quadriceps and hamstrings strength were obtained. Antagonist (hamstring) co-activation during knee extension was calculated as a ratio of hamstrings over quadriceps activity both during an isometric and during a functional sit to stand (STS) task (using kinematics) in groups of patients with extrapyramidal (n = 15), upper motor neuron (UMN) (n = 12), lower motor neuron (LMN) with (n = 18) or without (n = 12) sensory loss, primary muscle or neuromuscular junction disorder (n = 17) and in healthy matched controls (n = 32). Independent t-tests or Mann Witney U tests were used to compare between the groups. Correlations between variables were also investigated.

**Results:**

In healthy subjects mean (SD) co-activation of hamstrings during isometric knee extension was 11.8 (6.2)% and during STS was 20.5 (12.9)%. In patients, co-activation ranged from 7 to 17% during isometric knee extension and 15 to 25% during STS. Only the extrapyramidal group had lower co-activation levels than healthy matched controls (p < 0.05). Agonist isometric muscle strength and co-activation correlated only in muscle disease (r = -0.6, p < 0.05) and during STS in UMN disorders (r = -0.7, p < 0.5).

**Conclusion:**

It is concluded that antagonist co-activation does not systematically vary with the site of neurological pathology when compared to healthy matched controls or, in most patient groups, with strength. The lower co-activation levels found in the extrapyramidal group require confirmation and further investigation. Co-activation may be relevant to individuals with muscle weakness. Within patient serial studies in the presence of changing muscle strength may help to understand these relationships more clearly.

## Background

Muscle weakness can develop as result of infection, neurological problems, endocrine disorders, inflammatory conditions, rheumatologic diseases, genetic or metabolic conditions or may even be electrolyte or drug-induced [1]. Agonist muscle atrophy, failure of agonist muscle activation or excessive co-activation of antagonist muscle groups crossing the same joint may each in principle contribute to muscle weakness. Failure of agonist muscle activation (with or without secondary muscle atrophy) can be the result of neurological pathology at any level in the voluntary motor pathway but the extent to which co-activation processes are affected by pathology at different sites is unknown. Co-activation occurs during normal movement patterns and may improve movement efficiency during the performance of lower limb activities [2,3] with increased joint stabilization and protection. By contrast, excessive co-activation may result in impaired movement and weakness, particularly in the presence of neurological impairment [4].

Clinically increased muscle tone (e.g. spasticity in upper motor neuron syndrome, rigidity in Parkinsonism) might be expected to be associated with increased co-activation during voluntary muscle contraction. Co-activation has been quantified both during isometric muscle contractions [5,6], isokinetic contractions [7-9] and during the performance of functional activities  [2][10-12]. Most neurology based co-activation studies have however been undertaken in stroke patients or children with "cerebral palsy" where hypoxic ischaemic pathology relatively non selectively involves multiple CNS pathways and is associated with marked increases in muscle tone [13]. Such CNS involvement may however include pyramidal, para-pyramidal and extrapyramidal, cortical, subcortical and cerebellar structures as well as sensory and association pathways. It was therefore of interest to investigate patient groups with weakness due to different pathologies to see whether more selective patho-physiological causes of weakness were associated with differing levels of co-activation. Furthermore, it was unclear from the literature whether the level of co-activation systematically altered with the degree of weakness.

We hypothesised that antagonist co-activation would not be related to muscle strength per se but would be dependent on the site of the neurological lesion causing weakness. We expected that individuals with disorders of the extra-pyramidal and pyramidal systems would demonstrate higher levels of co-activation than healthy subjects. Co-activation of the hamstrings was studied both during isometric knee extension and a dynamic activity (sit to stand).

## Methods

### Study design

A between-subject design (case: control) was used. Five groups of patients (each n = 12 to n = 18) were compared to an age and sex matched control group from a pool of 32 of healthy subjects. Pilot study data suggested mean differences between neurology patients and healthy subjects of 8.3% for isometric co-activation, 55 N.m for quadriceps strength and 23.6 N.m for hamstrings strength. This equated to effect sizes of 1.38, 2.33 and 1.9 respectively. A sample size of 15 in each group would achieve a power of 0.94 with an α-level of 0.05 [14]. In the situation of lower numbers of cases being recruited e.g. n = 12, the equal allocation power was 80%, power increases were obtained by using unequal allocation of cases and controls [15].

### Subjects

Subjects were recruited from patients seen at the neurology clinics of the University Hospital Wales, Cardiff. The main inclusion criteria for the subjects with neurological deficits ('neurology patients') were that the individual: a) had a condition causing lower limb weakness or perceived weakness (usually bilaterally) diagnosed in one of the categories in table 1 by a specialist neurologist according to their clinical assessment and b) able to stand and walk for a short distance either independently or with crutches or another type of walking aid. The categories of neurological deficit (see Table 1) represented a spectrum of causes of neurological muscle weakness based on the recognised pathology of the diagnosed disorder.

**Table 1:** Specific categories along with the illustrative diagnoses, numbers in each group, mean age, gender and functional scores represented by the Rivermead Mobility Index (RMI) for each category

Category	Illustrative specific diagnoses	Mean (SD) age in years; Gender: male/female	Median (range) RMI	Control mean (SD) age in years; Gender: male/female
Primary muscle or neuromuscular junction disorder (n = 17)	Muscular dystrophy (n = 9) Polymyositis (n = 5) Myasthenic syndrome (n = 1) Acid-maltase deficiency (n = 1) Familial periodic paralysis (n = 1)	53.4 (12.4) 8 male, 9 female	12 (9 to 15)	51.7 (11.0) 8 male, 9 female (n = 17)
Peripheral nerve disorder with sensory loss (n = 18)	Guillain Barré syndrome (n = 9) Chronic inflammatory demyelinating polyneuropathy or sensory/motor neuropathy (n = 7) Axonal sensory/motor polyneuropathy (n = 1) Sensory peripheral polyneuropathy (n = 1)	56.7 (13.9) 7 male, 11 female	11.5 (4 to 15)	56.7 (11.0) 7 male, 11 female (n = 18)
Lower motor neuron (LMN) disorder with no or minor sensory loss (n = 12)	Motor neuropathy (n = 3) Motor neuron disease (clinical LMN signs only) (n = 3) Spinal muscular atrophy (n = 5) Lower motor neuron syndrome (n = 1)	52.9 (14.9) 10 male, 2 female	12 (4 to 15)	57.0 (13.5) 14 male; 9 female (n = 24)
Upper motor neuron lesions (UMN) (n = 12)	Hereditary spastic paraplegia Motor neuron disease with clinical UMN signs only (n = 1) Pyramidal Adrenoleukodystrophy (manifesting carrier) (n = 1)	51.6 (11.6) 7 male, 5 female	11.5 (7 to 14)	56.8 (14.5) 14 male; 9 female (n = 24)
Extra-pyramidal disorder (n = 15)	Parkinson's disease (PD) (n = 15)	64.3 (10.4) 11 male, 4 female	14 (9 to 15)	64.7 (9.5) 11 male, 4 female

A convenience sample of 32 healthy volunteers was recruited from local volunteer, charity and social groups to the study. This sample was sufficiently large enough to allow for matching of case to control in each of the 5 groups according to gender, age, height and weight. The main inclusion criteria for the healthy volunteers were that they were resident in the local vicinity and had no mobility restrictions or general health problems. Recruited volunteers were involved in a representative range of normal activities with none participating in elite sports activities.

The study was approved by the Bro Taf local research ethical committee. Subjects were required to provide informed written consent. In total, 74 neurology subjects who satisfied the inclusion criteria for the study were recruited to the study. Demographic details of each group are shown in Table 1.

### Functional ability

As a general measure of self reported mobility the Rivermead (RMI) mobility index score [16] was evaluated (see Table 1).

### Isometric strength

The strength of the right quadriceps and hamstrings muscles were evaluated using a KINCOM dynamometer (KINCOM 125E plus; Chattecx Corporation, Oxfordshire, OX6 0JX, UK). The subject being tested was seated with hips and knees flexed to 90°. The right leg was secured into an instrumented cuff positioned at a point approximately equi-distant between the knee and ankle joint (the moment arm was recorded and used in processing of strength data) with a stabilization strap across the thigh of the leg being tested. A seat belt was used to secure the subject in the sitting position and prevent them from altering the position during the data collection. The subjects were asked not to hold onto the chair with their hands during muscle contractions. They were required to initiate and maintain a maximal voluntary contraction for 5 seconds before relaxing. Verbal encouragement was given. The maximum force produced over 4 isometric contraction attempts was used for further analysis. A one minute resting period between each repetition of a muscle contraction was maintained.

The comparison between diagnostic groups (not matched for age, gender and weight) necessitated the use of predicted muscle strengths. To incorporate the confounding influence of gender, age, height and weight on muscle strength values, the mean absolute quadriceps and hamstring muscle strength in Newton metre (N.m) were expressed as a percentage of the mean predicted muscle strength in N.m. The predicted strength in kg was calculated using the National Isometric Muscle Strength Consortium regression equations [17] (right knee extension = (- (age * 0.38) + (sex * 18.44) + ((weight/height squared) * 0.62) + 34.41) and right knee flexion = (- (age * 0.16) + (sex * 8.78) + ((weight/height squared) * 0.08) + 22.47)). Gender was assigned a value of 1 for male and 0 for female. Thereafter, the predicted strength in kg was converted to strength in N.m by multiplying by 9.81 and the approximate moment arm for height according to published anthropometric data [18]. The moment arm was that of the distance between the knee and point of force application at the ankle as used in normative data collection protocol for the determination of the regression equations.

### Sit-to-stand (STS)

Subjects were asked to stand up, without the use of their arms for assistance if possible, from an armless, backless height adjustable chair (RH Support Froli; RH Form, London SW2 2AL, UK). The chair height was set to correspond to 100% of knee joint height to the floor of subject. The chair was placed on a force plate (Kistler 9253A12 Multi-component force plate; Kistler Instruments Ltd, Hampshire, GU34 2QJ, UK) whilst the subject's feet were placed on a second force plate situated adjacent to the first plate. Foot position was standardised to placement on this second force plate in an area of 40 centimetres (width) by 40 centimetres (depth) with variation in medio-lateral and anterior posterior placement of +/- 2.5 centimetres from the centre of the force plate permitted. This variation was necessary due to the nature of the included conditions; some individuals were unable to perform the task of STS without a marginal amount of flexibility in where they placed their feet. This allowed for a truer representation of the ways in which people with muscle weakness achieved a standing position. During STS, kinematics were obtained in the sagittal plane using the VICON 512 motion analysis system with reflective markers placed on the lower limbs of the subject being tested (VICON Motion systems, Oxford, OX2 OJB, UK). The phases of STS [33] were identified as follows; movement initiation was determined as the point when the trunk first started to lean forwards; the force plate under the chair was used to identify seat off as the time when it was fully unloaded. Kinetics during STS were calculated from the ground reaction force obtained from Kistler force plates. An inverse dynamic approach using a linked segment model of the human body was used to calculate the net knee moment during STS.

### Determination of co-activation

Surface EMG (SEMG) at rest during the maximum isometric voluntary contractions (MVC) and during STS was recorded at 1000 Hz (sampling frequency) for the quadriceps and hamstring muscles using an 8 cable telemetry system (Octopus; Bortec Electronics Inc., Alberta, Canada; amplifier input impedance: 10 GOhm; frequency response: 10–1000 Hz; common mode rejection ratio: 115 Db). Differential pre-amplifiers were used, which allowed for early suppression of noise and movement artifact in the raw signal [19,20]. Silver/silver chloride electrodes with a conductive area of 10 mm2 (Kendall Meditrace 230; Tyco Healthcare, Hampshire, UK) were applied to the right quadriceps (Vastus Medialis, Vastis Lateralis, Rectus Femoris) and hamstrings (Semi-Tendinosus, Biceps Femoris) of each subject according to the Surface electromyography for the non-invasive assessment of muscles (SENIAM) European recommendations for surface electromyography [21]. The raw SEMG signal for each muscle component was rectified and low pass filtered (digital Butterworth filter: 2nd order, bi-directional zero phase lag, 20 Hz cut-off frequency) to create a linear envelope for further analysis using Matlab 6.5 software (The MathWorks, Natick, MA). The SEMG signals were then averaged to provide a representative signal for each muscle group (quadriceps and hamstrings). The average SEMG activity in a 50 ms. epoch, associated with the maximum isometric strength, was calculated at the point of the maximal force achieved (incorporating a 50 ms. electromechanical delay representing the temporal delay between muscle electrical activity and realization of force). The same approach was used during STS to relate EMG activity to the maximal knee moment.

The net knee moment was considered the resultant of the agonist minus the antagonist (see Table 2, equation 1). For the net MVC extension moment this was the Quadriceps muscle moment minus the Hamstrings muscle moment (equation 2). The net MVC flexion moment was considered the Hamstring muscle moment minus the Quadriceps muscle moment (equation 3). The estimated Quadriceps muscle moment in both conditions was assumed to be represented by an unknown constant (a) multiplied by the EMG value for quadriceps (equation 4). Equally, the Hamstring muscle moment was assumed to be represented by an unknown constant (b) multiplied by the EMG value for hamstrings (equation 5). Estimated muscle moments were determined by solving for the constants (a) and (b) using two equations (2 & 3 in combination with 4 & 5) (one for extension and one for flexion) with two unknowns. The co-activation coefficient (equation 6) under isometric conditions was then calculated as the estimated moment of antagonist divided by the estimated moment of the agonist and multiplied by 100% to produce the percentage co-activation as used by for instance Ikeda et al. [5]. The estimated muscle moments were used in this equation to account for the difference in muscle mass. (Quadriceps femoris is approximately twice the size of the hamstrings and therefore much stronger).

**Table 2:** Equations used to calculate co-activation co-efficient

Net knee moment = moment (agonist) - moment (antagonist) (Eq. 1)
Extension knee moment = Quadriceps moment - Hamstrings moment (Eq. 2)
Flexion knee moment = Hamstrings moment - Quadriceps moment (Eq. 3)
Quadriceps muscle moment = constant (a) × Quadriceps EMG (Eq. 4)
Hamstrings muscle moment = constant (b) × Hamstring EMG (Eq. 5)
Co-activation=b×HMSEMGa×QCSEMG×100% (Eq. 6) MathType@MTEF@5@5@+=feaafiart1ev1aaatCvAUfKttLearuWrP9MDH5MBPbIqV92AaeXatLxBI9gBaebbnrfifHhDYfgasaacH8akY=wiFfYdH8Gipec8Eeeu0xXdbba9frFj0=OqFfea0dXdd9vqai=hGuQ8kuc9pgc9s8qqaq=dirpe0xb9q8qiLsFr0=vr0=vr0dc8meaabaqaciaacaGaaeqabaqabeGadaaakeaaieqacqWFdbWqcqWFVbWBcqWFTaqlcqWFHbqycqWFJbWycqWF0baDcqWFPbqAcqWF2bGDcqWFHbqycqWF0baDcqWFPbqAcqWFVbWBcqWFUbGBcqGH9aqpdaWcaaqaaiabbkgaIjabgEna0kabbIeaijabb2eanjabbofatjabbweafjabb2eanjabbEeahbqaaiabbggaHjabgEna0kabbgfarjabboeadjabbofatjabbweafjabb2eanjabbEeahbaacqGHxdaTcqWFXaqmcqWFWaamcqWFWaamcqWFLaqjcaWLjaGaaCzcamaabmaabaGaeeyrauKaeeyCaeNaeiOla4IaeeiiaaIaeGOnaydacaGLOaGaayzkaaaaaa@604A@

Since STS requires a net extensor moment at seat off, the quadriceps was assumed to be the agonist and hamstrings the antagonist during the calculation of co-activation during STS [22,23]. The co-activation during STS was obtained by applying the same constants (a & b) as obtained during the isometric calculation of co-activation at the point of the maximum net knee moment [5].

### Statistical analysis

Each group was compared with a control group matched on marginal distributions of means for age, height and weight. Inferential testing was completed using The Statistical Package for the Social Sciences (SPSS) version 11. Normality and equal variances of the data was assessed to allow for the appropriate choice of statistical test. Independent t-tests and in cases where normality was not shown, the non-parametric Mann Witney U test were used to compare between the 2 unrelated groups. In order to explore relations between co-activation and muscle strength, correlations between variables for the pooled healthy control subjects as well as the separate neurology patient groups were explored using a two-tailed Pearson's correlation co-efficient. Significance was established at 0.05 level.

## Results

### Functional ability

All patients tested in this study were able to walk 10 metres independently. RMI scores ranged from 4 to the maximal possible 15 across the diagnostic groups (see Table 1).

### Isometric strength

All patient groups had weaker knee flexors and knee extensors than matched healthy controls although this did not reach significance in the PD and LMN (without sensory loss) groups with respect to knee extension (see Table 3). The degree of weakness varied both within and between groups: for example patients with primary muscle disease were the weakest overall (see Table 3). Muscle strengths in the healthy control groups were close to the values predicted for both the quadriceps and hamstrings muscles equating to a mean (SD) absolute value of 152.3 (88.9) N.m for quadriceps and 82.2 (40.3) N.m for hamstrings muscles respectively.

**Table 3:** Mean (SD) muscle strength and co-activation variables across all diagnostic groups (* p ≤ 0.05; ** p ≤ 0.01 when compared to a matched control group)

Diagnostic groups	Mean (SD) (95% CI difference) predicted strength: quadriceps (%)	Mean (SD) (95% CI mean difference) predicted strength: hamstrings (%)	Mean (SD) (95% CI mean difference) isometric co-activation (%)	Mean (SD) (95% CI mean difference) co-activation during STS (%)
Muscle disease (n = 17)	50.6 (30.1) ** 36.9 to 88.7	55.9 (42.7) ** 32.7 to 85.4	17.4 (15.2) -1.2 to 15.1 ◊	22.3 (23.4) -18.5 to 7.9 ◊
LMN (sensory loss) (n = 18)	87.4 (27.5) -45.3 to 0.6	61.4 (22.4) ** 31.4 to 66.2	7.3 (5.1) -6.9 to 0.6 ◊	15.7 (11.0) -14.2 to 1.5
LMN (sensory intact) (n = 12)	53.4 (38.0) ** 21.5 to 78.3	55.6 (20.5) * 30.2 to 70.5	12.7 (10.9) -5.3 to 9.2 ◊	16.3 (10.6) Δ -12.8 to 6.5
UMN (n = 12)	67.2 (30.7) ** 12.1 to 65.4	55.9 (33.5) ** 19.4 to 67.1	9.4 (9.3) -7.7 to 2.9 ◊	24.7 (16.5) -7.5 to 13.6 ◊
Extra-pyramidal lesion (n = 15)	81.3 (36.4) -48.8 to 11.6	65.7 (30.6) * 13.9 to 57.0	6.7 (4.3) ** -11.2 to -3.9	14.7 (13.3) * -17.9 to 0.9 ◊
Control subjects (n = 32)	102.4 (37.0)	103.2 (30.1)	11.8 (6.2)	20.5 (12.9)

◊ non-parametric comparisons between groups were used hence, it is only possible to present approximate confidence intervals

Δ based on the means of data from 8 subjects. 4 subjects in this group used hip and trunk flexion strategies to achieve STS thus preventing calculation of co-activation at the point of the maximum knee extension moment.

### Co-activation

**Figure 1:**
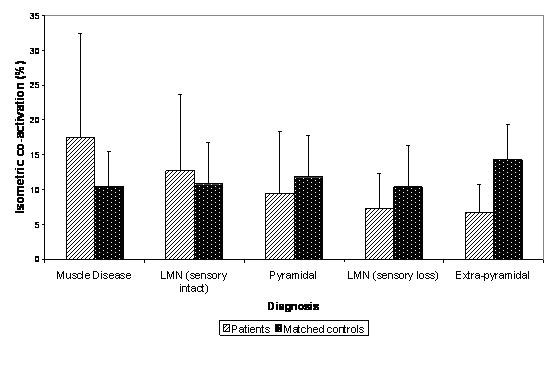
Isometric comparative mean hamstrings co-activation during quadriceps agonist activity across the included diagnostic groups (mean control group isometric co-activation was approximately 11%; represented by solid black line) (* p ≤ 0.05; ** p ≤ 0.01 when compared to a matched control group)

In healthy subjects mean (SD) co-activation of hamstrings during isometric knee extension was 11.8 (6.2)% and during STS was 20.5 (12.9)%: in neurology patient groups mean values for co-activation during isometric knee extension ranged from 7 to 17% (see Figure 1) and during STS from 15 to 25%. Levels of co-activation did not differ significantly between healthy and neurology groups either during isometric knee extension or during STS with the exception of the extra-pyramidal group who demonstrated significantly lower levels of co-activation (isometric (p < 0.01) and STS (p < 0.05)) than their matched healthy control group.

### Relationship between muscle strength and co-activation

In healthy subjects there were no correlations between isometric muscle strength and co-activation of hamstrings during knee extension under either isometric or STS conditions.

In neurology patients correlation analysis by diagnostic group showed a significant negative correlation between isometric quadriceps strength and co-activation of hamstrings during isometric knee extension only in muscle disease patients (r = -0.6; p < 0.05). A significant negative correlation was also identified between isometric quadriceps strength and co-activation during STS in the UMN group (r = -0.7; p < 0.05) but not in any other group (see Table 4).

**Table 4:** Relationships between isometric quadriceps muscle strength and co-activation identified using Pearson's correlation co-efficients (r) for each group (* p ≤ 0.05; ** p ≤ 0.01)

Diagnostic group	Extra-pyramidal	UMN lesions	LMN (sensory intact)	LMN (sensory loss)	Muscle disease
Isometric co-activation	r = -0.09	r = -0.3	r = -0.4	r = -0.1	r = -0.7 **
Co-activation during STS	r = -0.06	r = -0.7 *	r = 0.06	r = 0.4	r = -0.4

## Discussion

The present study aimed to investigate whether antagonist co-activation was related to muscle weakness and whether the degree of co-activation was different according to the site of the causative neurological lesion. Uniquely, co-activation was evaluated during both isometric contractions and a functional activity (sit-to-stand).

Although all patients tested were significantly weaker with respect to knee extensors and/or flexors when compared to an age, height and weight matched control group, there were some systematic strength differences between neurology diagnostic groups which potentially could be confounding factors in interpreting the findings of this study. The range of functional abilities was similar across diagnostic groups. It is important to note that a pragmatic approach of investigating the SEMG and isometric strength data only from the right leg of each individual was used. This was necessary as it was important that the subjects were not encumbered by numerous SEMG telemetry cables and fatigued by a lengthy data collection process requiring performance of functional activities that were challenging for many of the subjects. We did however collect the strength data bilaterally; there was no indication of major asymmetry of strength between sides and clinically there was no indication of a difference in diagnostic causation of weakness between right and left sides.

In healthy subjects, co-activation levels of hamstrings during isometric knee extension and co-activation during STS were similar to those previously reported. Co-activation during isometric quadriceps contraction has been found to range from 10.7 to 14.7% in 20 healthy sedentary males (mean (SD) age 22.1 (0.9))[24]. In 12 healthy control subjects (aged 25–59), antagonist hamstrings activity during a maximal isometric contraction of the quadriceps muscle was approximately 13% (+/- 5.8) [10]. During functional tasks such as standing up from a chair as well as sitting down and walking up and down stairs, hamstrings co-activation levels have previously been found to range from 17% to 25% [25].

In the patients tested in this study, co-activation levels across neurology groups were variable but comparable and mostly not different to that seen in healthy subjects (co-activation ranged from 7 to 17% during isometric knee extension and 15 to 25% during STS). This is similar to what has been seen in the literature, for example co-activation in stroke patients during an isometric quadriceps maximal contraction was found to be 14.2% (+/- 7.3) [10] and 12.2% (+/- 14.4%) during knee extension in children with cerebral palsy [5].

Interestingly, neither the presence of an "upper motor neuron" syndrome nor the presence of sensory impairment alongside weakness appeared to systematically result in increased co-activation above levels seen in healthy subjects. A range of studies have assessed co-activation in people with stroke, PD, spinal cord injury and in children with cerebral palsy [26-28]. We are not aware of studies which have measured levels of co-activation in a wide range of diagnostic categories or in individuals with peripherally mediated weakness or sensory impairment. Comparisons with healthy subjects are also not readily apparent. Of potential relevance to these findings is the large variation within each subject group for the co-activation measures. Differences may not have been detected due to insufficient observed statistical power. Observed effect sizes (ranging from 0.24 to 0.96 for each group and their matched control group) were substantially lower than that used for the initial power calculations. Further investigation would be required using larger numbers of participants to confirm or refute these non-significant findings.

Unexpectedly, Parkinson's disease (PD) patients demonstrated significantly lower co-activation levels (both isometric and during STS) when compared to a matched healthy control group. Patients with PD experience difficulty in initiation of movements that has been attributed to bradykinesia, muscle weakness and excessive co-activation as well as the clinical feature of limb rigidity [23,29]. Rigidity gives rise to muscular stiffness with clinical hypertonicity in agonist and antagonist muscle groups on passive movement [30] suggesting intuitively that higher levels of co-activation might be anticipated. Selective weakness did not explain these lower levels of co-activation since the level of force produced during the isometric hamstring test (agonist) was well above that generated during co-activation (antagonist) activity. It is possible that the reduced co-activation identified is linked with the benefits of the medication used to treat PD, however this study was not specifically designed to investigate medicated versus non-medicated patients as all subjects were tested in the 'on phase' of medication. This may be worthy of sequential study within individuals on and off medication.

Overall there was some evidence for a link between increasing weakness and increasing level of co-activation in muscle disease patients during isometric knee extension and in patients with UMN lesions during STS. Co-activation could critically contribute to a reduction of net agonist force output in such disorders and in UMN lesions muscle activation during weight bearing might be influenced by altered stretch reflex sensitivity. However the data requires independent confirmation as conceivably altered kinematics of STS and/or the range of compensatory strategies used by neurology patients could have influenced the data. Exploration of relationships between co-activation, kinematic and kinetic characteristics of STS did not however reveal any significant correlations.

Patients who were very weak and/or unable to walk the required distance and hence complete the testing protocol were excluded from this study and so the lowest end of the muscle strength spectrum is not represented. Further exploration across a range of diagnostic groups with specific reference to very weak individuals or serial investigations of patients recovering from severe weakness (e.g. Guillain-Barré syndrome) may be of interest in considering whether co-activation critically limits net agonist activity and joint movement.

In conclusion, co-activation levels did not appear to vary systematically between diagnostic neurology groups when compared to healthy subjects with the possible exception of extra-pyramidal disorder where co-activation tended to be lower both during isometric and STS conditions. Secondly, co-activation did not systematically vary according to muscle strength in healthy subjects or in neurology patient groups during two activities (isometric knee extension and STS) except in muscle disease (isometric) and UMN lesions (STS) where there was an indication of increasing co-activation with increasing weakness.

The study demonstrates approximately 10% co-activation of hamstrings during knee extension in both healthy individuals and in neurology patients during isometric quadriceps contractions and 20% during STS which overall remains fairly stable in the presence of neurological disease. We suggest that co-activation should be taken into account in evaluating net agonist strength and potentially may be an element which can be manipulated therapeutically to improve function. Within-patient serial studies in the presence of changing muscle strength may help to understand the role of co-activation more clearly.

## Competing interests

The author(s) declare that they have no competing interests.

## Authors' contributions

RVD and CMW conceived of the study, and participated in its design and coordination and helped to draft the manuscript. MB participated in the design, recruitment of subjects, acquisition of data, analysis and interpretation of data; all authors read and approved the final manuscript.
